# Imaging diagnosis and efficacy monitoring by [^89^Zr]Zr-DFO-KN035 immunoPET in patients with PD-L1-positive solid malignancies

**DOI:** 10.7150/thno.87243

**Published:** 2024-01-01

**Authors:** Huihui He, Xiaowei Qi, Haitian Fu, Jianfeng Xu, Qihuang Zheng, Liping Chen, Yu Zhang, Haiying Hua, Wenhuan Xu, Zhenyu Xu, Xiaoping Chen, Qingjun You, Jianguo Lin, Gang Huang, Yong Mao, Chunjing Yu

**Affiliations:** 1 Department of Nuclear Medicine, Affiliated Hospital of Jiangnan University; Wuxi, China.; 2 Department of Pathology, Affiliated Hospital of Jiangnan University; Wuxi, China.; 3 Dongcheng AMS Pharmaceutical Co., Ltd.; Nanjing, China.; 4 Center of Radiological Imaging, College of Medicine, Indiana University, Indiana, USA.; 5 Department of Oncology, Affiliated Hospital of Jiangnan University; Wuxi, China.; 6 Institute of Oncology, Affiliated Hospital of Jiangnan University; Wuxi, China.; 7 NHC Key Laboratory of Nuclear Medicine, Jiangsu Key Laboratory of Molecular Nuclear Medicine; Jiangsu Institute of Nuclear Medicine, Wuxi, China.; 8 Shanghai Key Laboratory of Molecular Imaging, Shanghai University of Medicine and Health Sciences; Shanghai, China.

**Keywords:** PD-L1 antibody, tumor immunotherapy, efficacy monitoring, molecular imaging, PET

## Abstract

**Rationale:** Although programmed death-ligand 1 (PD-L1) inhibitors have achieved efficacy in cancer therapy, their response rate is low. Differences in the prognosis of patients with cancer under anti-PD-L1 treatment are related to the PD-L1 level in tumors. Accurate PD-L1 detection can optimize the accuracy of tumor immunotherapy and avoid ineffective clinical diagnosis and treatments.

**Methods:** We investigated the imaging efficiency and therapy monitoring capacity of [^89^Zr]Zr-DFO-KN035 immunoPET for tumors. We labeled the monodomain anti-PD-L1 antibody KN035 with the radionuclide zirconium-89 and used this tracer for PET imaging. [^89^Zr]Zr-DFO-KN035 uptakes in patients with PD-L1-positive tumors, including primary and metastatic tumors, as well as in normal tissues, were comparatively assessed by using positron emission tomography/computed tomography imaging.

**Results:** In PD-L1-positive patients, [^89^Zr]Zr-DFO-KN035 was sensitive in tumor-targeting imaging and could detect multiple metastatic foci, including multiple bone metastases (tumor-to-muscle ratios of 7.102 and 6.118 at 55 and 120 h, respectively) and lymph-node metastases (tumor-to-muscle ratios of 11.346 and 6.542 at 55 and 120 h, respectively). The needed radioactive dose of [^89^Zr]Zr-DFO-KN035 (55.5-92.5 MBq) used in this study was considerably lower than that of [^18^F]FDG (370-555 MBq). [^89^Zr]Zr-DFO-KN035 monitored and predicted the site of adverse reactions in antitumor immunotherapy. Moreover, after antitumor treatment, [^89^Zr]Zr-DFO-KN035 enabled observational imaging for therapeutic efficacy evaluation, which can help predict patient prognosis.

**Conclusion:** [^89^Zr]Zr-DFO-KN035 can be used for the diagnosis and therapy monitoring of PD-L1-positive tumors and provide noninvasive and comprehensive observations for tumor diagnostic imaging, prognosis prediction, and efficacy evaluation.

## Introduction

As a novel antitumor therapy, tumor immunotherapy is attracting considerable attention and extensive research worldwide. Under the tumor immunotherapy strategy, various monoclonal antibody drugs are being developed and marketed to eliminate tumor cells by suppressing tumor immune-checkpoint signaling pathways and reactivating antitumor immune responses. Immunotherapy has achieved substantial improvement in the overall survival of patients with cancer 1-3. Tumor immunotherapy, which is suitable for almost all types of malignant tumors, has several advantages over traditional therapy. Clinical studies show that inhibiting tumor immune-checkpoint proteins exerts a certain effect on various cancers. The efficacy duration of tumor immunotherapy drugs *in vivo* is remarkably longer (single dose) than that of traditional chemotherapy, radiotherapy, and targeted therapy. Given its high specific efficacy, tumor immunotherapy may achieve a better degree of acceptance by patients than other therapies 4, 5. Blocking the PD-1/PD-L1 signaling pathway reportedly has an antitumor effect on numerous solid malignancies 6. PD-1/PD-L1 inhibitors have advantages as new immunotherapy agents for solid tumors, and research has verified that the combination of chemotherapy drugs with anti-PD-L1 drugs increases the significance of the therapeutic effect 7, 8. Therefore, how to make the best use of PD-1/PD-L1 inhibitors for suppressing immune-checkpoint signaling is bound to become a research hotspot in cancer immunotherapy.

Anti-PD-1/PD-L1 drugs currently on the market include anti-PD-1 antibodies (e.g., nivolumab, pembrolizumab, triplezumab, and sintilimab) and anti-PD-L1 antibodies (e.g., atozumab, durvalumab, and avelumab). Clinical trials on several anti-PD-L1 antibody drugs are ongoing. Therapy based on blocking the PD-1/PD-L1 signaling pathway has achieved encouraging therapeutic effects in the treatment of cancers. However, its overall response rate without cancer pre-screening remains low 9, 10. Accurate diagnosis is a prerequisite for accurate treatment. Further studies have shown that the difference in the prognosis of patients with cancer receiving anti-PD-1/PD-L1 therapy is related to PD-L1 expression at cancer sites, and the benefit of immunotherapy is more obvious in patients with high PD-L1 expression than in those without 10, 11. Therefore, the accurate detection of PD-L1 expression can be used to screen out patients who are highly sensitive to targeted anti-PD-1/PD-L1 immunotherapy, greatly favoring the optimization of the accuracy of tumor immunotherapy and avoiding ineffective clinical diagnosis and treatment decisions. As an integral part of individualized tumor therapy, the concomitant detection of PD-L1 plays an important role in the clinical pathological diagnosis for effective antitumor immunotherapy.

The immunohistochemical method is the only validated and routinely conducted methodology for evaluating PD-L1 expression level in clinical practice. It can reveal the binding sites of tumor antigen and specific antibodies in tissues and cells. However, this method cannot provide noninvasive, real-time, and comprehensive detection results. PD-1/PD-L1 immunotherapy may cause immune pneumonitis, skin rash, thyroiditis, and other adverse reactions 12-14, whereas immunohistochemistry (IHC) cannot enable the mechanistic prediction and monitoring of the occurrence of these side effects. IHC examination also has the following limitations: First, collecting pathological specimens, especially for deep tumors that cannot be biopsied, from some patients, such as those who refuse invasive biopsy, is difficult. Second, IHC can reflect only the expression of PD-L1 expression in tumor tissues at one time-point and cannot be monitored continuously to obtain the change in PD-L1 expression with time. Third, IHC may not reflect the actual PD-L1 expression in all malignancies due to the influence of a series of uncertain factors, such as sampling and specimen handling. Therefore, determining how to more scientifically and reliably screen patients who may benefit from immunotherapy or combination therapy scientifically and reliably is worthy of further study.

Molecular-imaging technology with radionuclide-labeled tracers enable real-time observation and have high specificity and high resolution as well as repeatable detectability without inflicting trauma 15, 16. Immuno-positron emission tomography (immunoPET), which combines the superior targeting specificity of monoclonal antibodies (mAbs) and the inherent sensitivity of positron emission tomography (PET), is gradually changing the theranostic landscape of several types of malignancies 17. ImmunoPET provides a noninvasive and whole-body visualization of *in vivo* immune checkpoint biodistribution, which may serve as a robust biomarker for predicting and monitoring responses to immune checkpoint inhibitors 18. Niemeijer et al. have achieved the whole-body PET/CT imaging of PD-1 expression in 13 patients with advanced NSCLC by using ^89^Zr-nivolumab, prior to treatment with nivolumab 19. As the first anti-PD-L1 monodomain antibody drug for tumor immunotherapy in clinical research and the first immunotherapy antibody drug adopting subcutaneous injection 20, KN035 has shown good safety and effectiveness in preclinical research and phase II/III clinical trials for various tumors 21, 22. Previous preclinical experiments have confirmed the targeting of [^89^Zr]Zr-DFO-KN035 in various animal models 21, 23. Furthermore, the efficacy of targeted antitumor drugs in xenograft mice can be dynamically observed with [^89^Zr]Zr-DFO-KN035 24.

In the present study, KN035 was labeled with the radionuclide zirconium-89 and PET/CT scanned *in vivo* to investigate the imaging efficiency and efficacy monitoring capacity of [^89^Zr]Zr-DFO-KN035 PET in patients. The uptake of [^89^Zr]Zr-DFO-KN035 in patients with PD-L1-positive solid tumors, including primary and metastatic cancers, as well as in normal tissues, were assessed by using PET/CT imaging. We expected to observe intuitively the specific targeting and retention of [^89^Zr]Zr-DFO-KN035 in PD-L1-positive tumors and the alterations in tumor uptake after treatment. Our results can help clarify the pharmacokinetic parameters of the KN035 molecule *in vivo* and guide follow-up clinical research on KN035-based drugs.

## Materials and Methods

### Study subjects

This work is a single-armed open study, and 12 patients with solid malignancies were enrolled. A microdose exploratory clinical trial of [^89^Zr]Zr-DFO-KN035 was conducted. All patients were diagnosed with cancer through routine histopathologic or cytological examination and specifically identified as positive or negative for PD-L1 via immunohistochemistry. The available solid tumor cases included, but were not limited to, lung primary cancer, vascular lymphoma, hepatocellular carcinoma, colorectal cancer, nasopharyngeal cancer, pancreatic cancer, neuroendocrine tumor, and melanoma. This study was approved by the Medical Ethics Committee of Affiliated Hospital of Jiangnan University. All procedures involving human participants or samples were performed in accordance with the ethical standards of the Independent Ethics Committee of Affiliated Hospital of Jiangnan University and the 1975 Declaration of Helsinki as revised in 2013. All patients were informed, consented before admission, and signed the informed consent form for their participation. This work is a registered clinical trial study 25.

### Safety monitoring and follow-up

Safety monitoring and follow-up were performed on all patients. Each patient was monitored for vital signs (body temperature, pulse, blood pressure, and respiratory rate) 30 min before the injection of the radioactive drug, 30 min (± 5 min) and 24 h (± 30 min) after injection, and 30 min before PET imaging acquisition at each time point. After the final scan, the subject was examined on the basis of physical and vital sign examinations, blood and urine routine assays, blood biochemistry and 12-lead electrocardiography. Each subject then completed 26-30 days of telephone interviews for the collection of security information. Furthermore, 3 out of 11 patients (No.1, 3, and 4) underwent [^89^Zr]Zr-DFO-KN035 PET/CT scanning at baseline and post-anti-PD-L1 treatment. Relevant indicators, such as serum tumor biomarkers, improvement in serum biochemical indicators, and reduction in concurrent symptoms, were followed up to evaluate the tumor burden after treatment.

### Administration of the radioactive tracer

The recombinant humanized PD-L1 single-domain antibody Fc fusion protein (KN035 for short) was supplied by Suzhou Alphamab Co. Ltd. [^89^Zr]Zr-DFO-KN035 was radiolabeled in accordance with the following steps: KN035 was conjugated with p-SCN-Bn-Deferoxamine by using a method previously reported method 21. In short, KN035 was diluted in 0.1 mol/L Na_2_CO_3_ (pH 7.2), added with DFO-Bz-NCS in DMSO, incubated for 60 min at room temperature, and purified by using PD-10 (Sephadex™ G-25 Medium). DFO-Bz-NCS-KN035 was labeled with the radionuclide zirconium-89 to form the injectable [^89^Zr]Zr-DFO-KN035 used in this study. The radiochemical purity and molar activity of the product were qualified with the control standard (> 95%) (**Figure S1**). The product had the molar activity of 2.95 MBq/μmol and specific activity of 37 MBq/mg. The molecular weight of KN035 was 79.6 kDa, and that of [^89^Zr]Zr-DFO-KN035 was approximately 80.4-81.2 kDa. Patients who met the inclusion criteria were enrolled in the study and given subcutaneous injections of 10 mg of [^89^Zr]Zr-DFO-KN035 at radioactive doses ranging from 59.2 MBq to 88.8 MBq after safety monitoring. [^18^F]FDG was administered intravenously (399.6-562.4 MBq).

### PET/CT imaging

After prophase safety monitoring, the patients were injected with the radiopharmaceutical [^89^Zr]Zr-DFO-KN035 at the set dose and then subjected to static scanning with whole-body PET/CT imaging. The first enrolled subject underwent PET/CT imaging at the set time points of approximately 24, 56, and 120 h after [^89^Zr]Zr-DFO-KN035 injection. The timing of the PET/CT scanning of the following subjects was adjusted in accordance with the results of the first subject. A clinical PET/CT system (Biograph 64, Siemens Inveon) was used, and the scanning time was recorded. The scanning time was set as static PET scanning for 10 min with the scanning energy window of 350 keV. PET/CT image reconstruction was performed after scan-data collection. The reconstruction algorithm used was 3D OSEM PSF, and the number of iterations was five. The image-processing software PMOD was used to process the images after reconstruction and data. The radioactivity values of the areas of interest and standard radioactive uptake values (SUVs) of different tissues and tumor sites were obtained. Furthermore, 5 out of 11 patients (No.1, 2, 3, 4, and 11) underwent usual [^18^F]FDG PET/CT scanning.

### Immunohistochemistry

IHC was used to identify and determine PD-L1 expression in the pathological sections of patients with tumor. Tumor tissues were fixed with 4% paraformaldehyde solution for 1 week twice. After being dehydrated in 30% sucrose, the tissue blocks were embedded in paraffin and cut into slices with a thickness of 5 μm. The tissue slices were blocked with 3% BSA in PBS and incubated at a concentration with 2 μg/ml primary antibody (anti-PD-L1 [E1L3N] rabbit monoclonal antibody; Cell Signaling Technology, #13684, Boston, MA, the USA) at 4 °C overnight. After being washed, HRP-conjugated goat anti-rabbit IgG secondary antibody (Beyotime Biotechnology, Shanghai, China; 1:500 dilution in volume) was incubated for 1 h at room temperature and washed with PBS three times. Slices were subjected to coloration and washing, mounted onto glass slides, sealed with 30% glycerin, and visualized under an inverted microscope (Olympus IX71, Japan). The tumor proportion score (TPS) is the percentage of tumor cells with PD-L1 membrane staining. TPS < 1% was considered as negative expression, whereas TPS ≥ 1% was defined as positive expression, wherein TPS 1%-49% and TPS ≥ 50% were defined as low expression and high expression levels, respectively.

### Statistical analysis

Data analysis was performed by using SPSS 18.0 software (SPSS Inc., the USA). Quantitative data, such as SUVs, were statistically presented in the form of mean ± SD. Paired sample t-test and one-way ANOVA were used for comparisons. Spearman analysis was utilized for linear correlation analysis. Differences at the inspection level of p < 0.05 were considered as statistically significant.

## Results

### Characteristics of enrolled patients

We screened all eligible patients with cancer who were admitted to the Department of Oncology of our hospital from March 2021 to November 2021. We enrolled 12 patients who met the admission criteria and signed an informed consent form. One of these cases had incomplete PET/CT scans and other information and was excluded from further investigation (**Figure S2**). Information on the basic characteristics of the remaining 11 enrolled cases is shown in **Table 1**. In accordance with the criteria for PD-L1-positive (TPS > 1%) and PD-L1 high-expressing cases (TPS > 50%) based on histopathological examination 26, 27, one case was negative for PD-L1, and the other 10 cases were positive for PD-L1, with four being PD-L1 high-expressing cases.

### [^89^Zr]Zr-DFO-KN035 immunoPET was useful compared with [^18^F]FDG for tumor detection in PD-L1-positive patients

**Figure 1:**
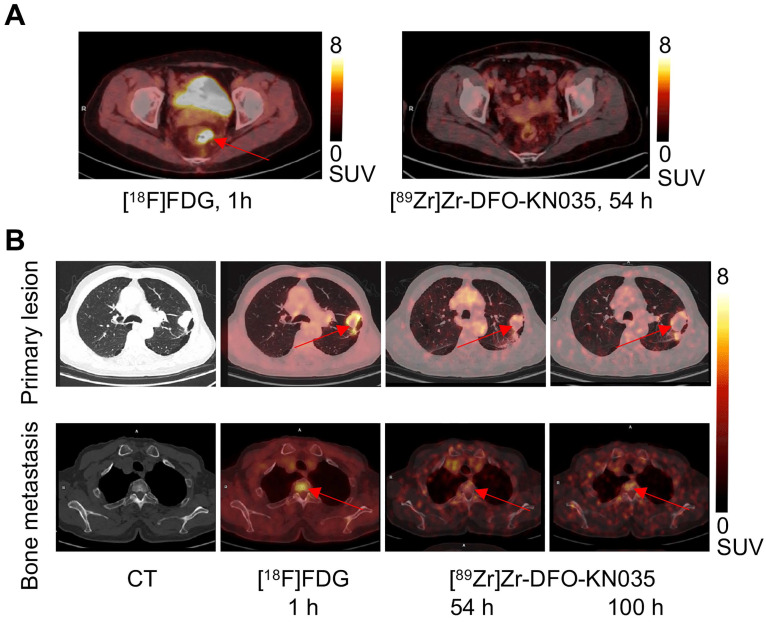
** Comparison of the [^18^F]FDG and [^89^Zr]Zr-DFO-KN035 PET imaging of cases negative (No. 11) and positive (No. 2) for PD-L1. (A)** Representative PET/CT imaging of transverse section of colorectal cancer in the same PD-L1-negative patient acquired by using [^18^F]FDG (1 h) and the PD-L1-targeting molecular-imaging tracer [^89^Zr]Zr-DFO-KN035 (54 h). **(B)** Representative CT and PET/CT images of layers of lung primary cancer (top lane) and one bone metastasis site (bottom lane; thoracic vertebrae 2) acquired by using [^18^F]FDG (1 h) and [^89^Zr]Zr-DFO-KN035 (54 and 100 h) in a PD-L1-positive patient. Red arrows indicate the locations of primary cancer and bone metastatic site.

The comparison of the [^89^Zr]Zr-DFO-KN035 and [^18^F]FDG PET imaging of patients with PD-L1-negative and -positive cancer patients indicated that the KN035 antibody had good specific targeting for the PD-L1 molecule *in vivo*. As shown in **Figure 1A and Figure S3**, [^89^Zr]Zr-DFO-KN035 immunoPET (scanned at 54 h after tracer injection) revealed no tumor-imaging features in a patient with PD-L1-negative (TPS < 1%) colorectal cancer and liver metastasis. In a patient with PD-L1-positive primary lung cancer and multiple bone metastases (TPS = 40%) and at a lower radioactive dose than [^18^F]FDG PET/CT (540.2 MBq, 1 h), [^89^Zr]Zr-DFO-KN035 immunoPET (85.1 MBq, 54 h) demonstrated sensitive tumor-specific imaging and revealed numerous metastatic lesions, such as multiple bone metastases (**Figure 1B**).

The numbers of primary and metastatic tumor lesions found through [^18^F]FDG (*n* = 5 patients) and [^89^Zr]Zr-DFO-KN035 (*n* = 10 patients) PET imaging are presented in **Table 2**. No differences existed between the average lesion numbers in PET imaging with the two tracers (p > 0.05 for primary, lymph node (LN) metastatic and bone metastatic lesions). As shown in **Table 3**, the uptake of [^89^Zr]Zr-DFO-KN035 (56 h) in the primary tumor lesions of the 10 PD-L1-positive patients were relatively high (target-to-muscle ratio of SUVmax for 2.10-20.50 and target-to-muscle ratio of SUVmean for 1.49-10.42). However, [^89^Zr]Zr-DFO-KN035 tumor uptake (R = -0.019 and -0.031) and the visualized metastatic lesion number (R = 0.45) did not reveal significant linear correlation with tumor PD-L1 expression on the basis of the IHC assay score.

**Figure 2:**
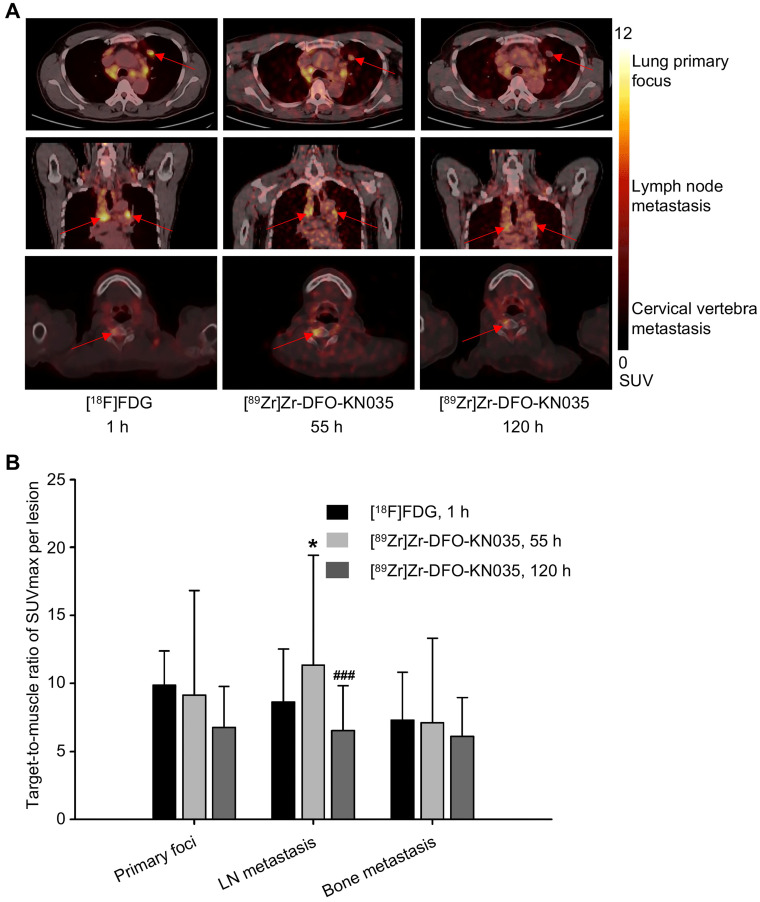
** Radioactive substance uptakes in lung primary tumors and metastatic lesions in PET imaging with two tracers. (A)** Representative **(Case No. 1)** PET imaging results obtained with [^18^F]FDG and [^89^Zr]Zr-DFO-KN035 in lung primary tumor foci, lymph node metastatic lesions and bone metastatic lesion (cervical vertebra). Red arrows indicate the locations of primary cancer, lymph node metastatic lesions, and bone metastatic sites. **(B)** Statistical results of the target-to-muscle ratios of the SUVmax of each lesion in patients. Data are expressed as mean ± SD. One-way ANOVA was used. *p < 0.05 vs. [^18^F]FDG and ^###^p < 0.001 vs. [^89^Zr]Zr-DFO-KN035 at 55 h. Scale bar of SUV 0-12 for [^18^F]FDG and [^89^Zr]Zr-DFO-KN035.

Representative PET images with [^18^F]FDG (1 h) and [^89^Zr]Zr-DFO-KN035 (55 and 120 h) showing primary (lung primary focus), LN metastatic, and bone metastatic lesions (cervical vertebra) are presented in **Figure 2A**, respectively. The statistical results of the target-to-muscle ratios (TBRs) of the SUVmax of each lesion are provided in **Figure 2B** (*n* = 5 and 10 for primary foci, 29 and 46 for LN metastases, 9 and 19 for bone metastases; F_[2,22]_ = 0.673, p = 0.520 for primary lesions; F_[2,118]_ = 8.004, p = 0.001 for LN lesions; and F_[2,44]_ = 0.280, p = 0.757 for bone lesions). This finding suggested that LN metastases had higher radioactive uptake in [^89^Zr]Zr-DFO-KN035 immunoPET at 55 h than in [^18^F]FDG PET/CT at 1 h (p = 0.046) and that the uptake of [^89^Zr]Zr-DFO-KN035 was sustained well even at 120 h post-injection but then dropped significantly (p < 0.001).

### Comparative study of the diagnostic efficiency of [^89^Zr]Zr-DFO-KN035 immunoPET and [^18^F]FDG PET in patients with PD-L1-positive lung cancer

**Figure 3:**
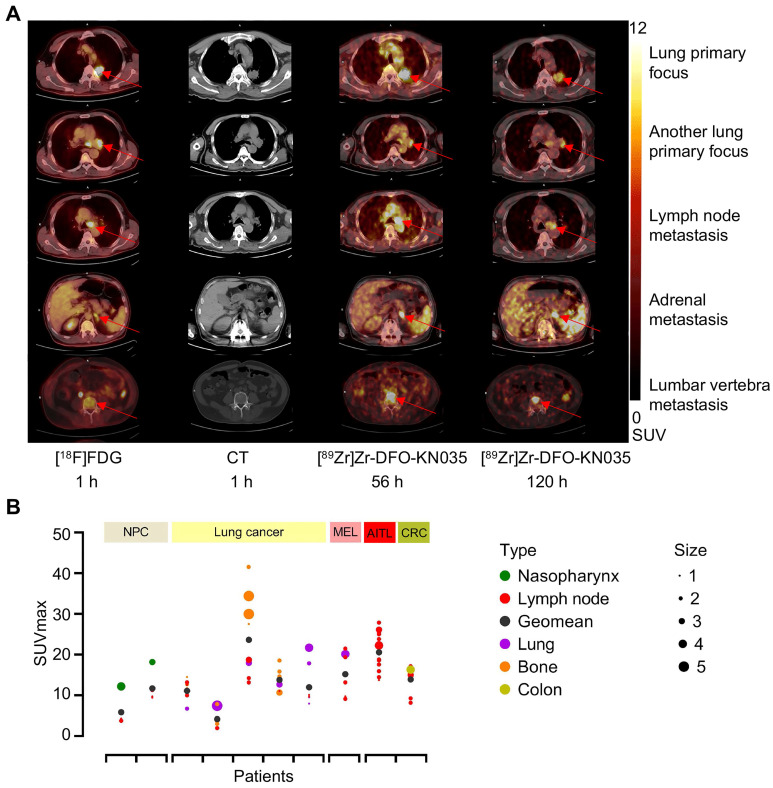
** Comparison of two PET/CT methods for the imaging diagnosis of primary lung cancer and multiple metastases in PD-L1-positive patients. (A)** Representative **(Case No. 3)** CT and PET/CT images of a PD-L1-positive patient with lung primary cancer and multiple bone metastases. The images were acquired before antitumor treatment by using [^18^F]FDG (1 h) and [^89^Zr]Zr-DFO-KN035 (56 and 120 h). Red arrows indicate the locations of primary cancer, lymph node metastatic lesions, and bone metastatic sites. **(B)** Overview statistical graph of [^89^Zr]Zr-DFO-KN035 (56 h) uptake (SUVmax) in different primary and metastatic tumors of 10 patients with PD-L1 positive cancer before treatment. Cases were grouped in accordance with primary tumor types (NPC, nasopharynx cancer, 2 cases; lung cancer, 5 cases; MEL, melanoma, 1 case; lymphoma, Angioimmunoblastic T-cell lymphoma (AITL), 1 case; CRC, colorectal cancer, 1 case). Type, primary and metastatic tumor types. Size, tumor sizes based on long diameters (1, 0-1 cm; 2, 1-2 cm; 3, 2-3 cm; 4, 3-4 cm; 5, 4-5 cm).

In patients with PD-L1-positive lung cancer, as shown by the different cross-sectional images in **Figure 3A**, [^89^Zr]Zr-DFO-KN035 immunoPET can detect numerous metastatic lesions, such as bone, LN, and adrenal metastases, and [^89^Zr]Zr-DFO-KN035 had uptakes and high TBRs at numerous tumor sites. **Figure S4A** shows the statistical results of [^18^F]FDG (1 h) and [^89^Zr]Zr-DFO-KN035 immunoPET (56 and 120 h) uptakes in regions of interest (ROIs) (SUVmean, *n* = 3 for each region and each time point). As shown in **Figure S4B**, the TBRs of the SUVmean of [^18^F]FDG and [^89^Zr]Zr-DFO-KN035 in different regions (*n* = 3 per site) were similar. However, [^89^Zr]Zr-DFO-KN035 immunoPET had a slightly higher TBR than [^18^F]FDG for lung primary cancer foci ([^18^F]FDG, 8.798 ± 3.417; [^89^Zr]Zr-DFO-KN035 120 h, 12.448 ± 1.745; F_2,6_ = 0.404, p = 0.048) and multiple bone metastases (cervical vertebra: FDG, 7.010 ± 2.178; KN035 120 h, 17.561 ± 6.282; F_2,6_ = 1.638, p = 0.043 and sacral vertebra: FDG, 4.357 ± 0.877; KN035 120 h, 18.026 ± 8.109; F_2,6_ = 1.270, p = 0.044). **Figure 3B** presents a statistical summary of the primary tumor classification, metastatic tumor type, tumor size (long diameters), and [^89^Zr]Zr-DFO-KN035 (56 h) radioactive uptakes (SUVmax) before treatment of all 10 PD-L1-positive patients.

### Therapeutic efficacy observation by [^89^Zr]Zr-DFO-KN035 immunoPET in patients with PD-L1-positive tumors

**Figure 4:**
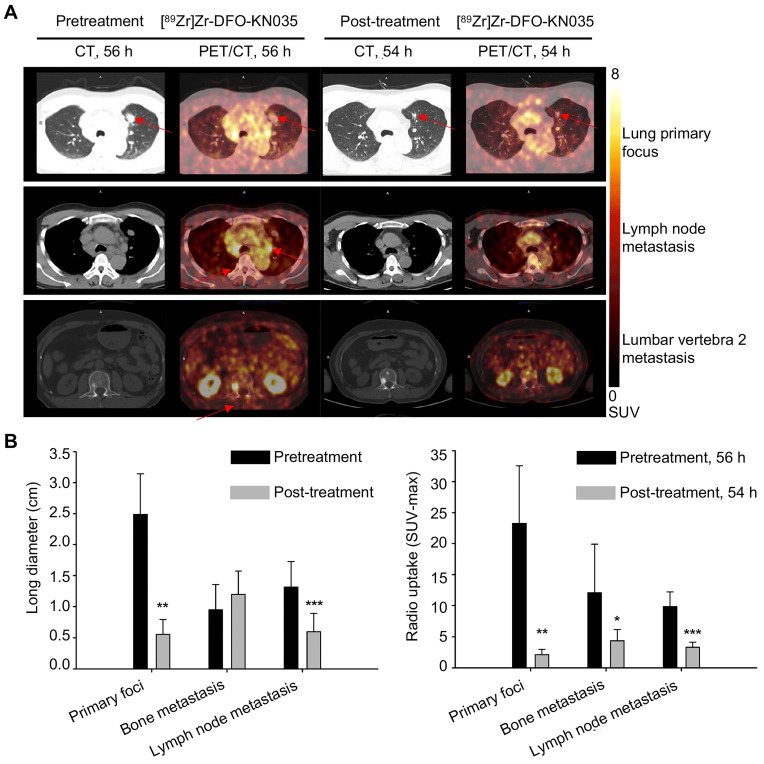
** Comparison of primary tumors and metastases in PD-L1-positive patients using [^89^Zr]Zr-DFO-KN035 immunoPET before and after treatment. (A)** Representative **(Case No. 1)** CT and PET/CT images of the layers of primary focus (first row), lymph node metastasis (second row), and bone metastasis (third row, lumbar vertebra 2) sites in a patient with PD-L1-positive cancer (lung primary cancer with bone, lymph node, and brain metastasis) acquired by using [^89^Zr]Zr-DFO-KN035 tracer before (56 h) and after (54 h) antitumor therapy. Red arrows show the locations of primary tumor and metastasis focus sites. **(B)** Statistical chart and comparison of the long diameters and the radioactive uptake values (SUVmax) of the primary and metastatic tumors in PET molecular imaging with [^89^Zr]Zr-DFO-KN035 before (56 h) and after (54 h) treatment. Data are expressed as the mean ± SD, and *n* = 3-7 for each site. Paired samples t-test was conducted. *p < 0.05, **p < 0.01, and ***p < 0.001 vs. pretreatment group.

Three sections, including lung primary foci, LN metastases, and bone metastases (lumbar vertebra 2 metastasis) are shown in **Figure 4A** to compare PET/CT imaging at the scanning time-points of 56 h pretreatment and 54 h post-treatment in a patient with PD-L1-positive lung cancer. **Figure S5A** illustrates the comparison of the PET/CT imaging of the same corresponding sections of the patient with lung cancer before (120 h) and after treatment (120 h). PET/CT results revealed that the tumor lesion volumes (represented by long diameters) at multiple sites after treatment altered when self-compared with those before treatment. As summarized in **Figure 4B**, the primary tumor foci significantly shrunk (*n* = 3; pretreatment, 2.490 ± 0.657 cm; post-treatment, 0.557 ± 0.240 cm; t = 5.520, p = 0.012), and LN metastatic foci significantly reduced (*n* = 7; pretreatment, 1.315 ± 0.411 cm; post-treatment, 0.600 ± 0.297 cm; t = 8.187, p < 0.001). Conversely, multiple bone metastases presented no significant changes in volume (*n* = 7; pretreatment, 0.953 ± 0.405 cm; post-treatment, 1.197 ± 0.376 cm; t = 1.691, p = 0.142). The statistical data of the [^89^Zr]Zr-DFO-KN035 immunoPET results provided in **Figure 4B and Figure S6B** show that the average radioactive uptakes (SUVmax) of multiple tumor foci significantly decreased after antitumor treatment. The radioactive uptakes of [^89^Zr]Zr-DFO-KN035 in primary tumor foci (*n* = 3; 56 h pretreatment, 23.280 ± 9.293; 54 h post-treatment, 2.155 ± 0.843; t = 6.387, p = 0.009; 120 h pretreatment, 18.242 ± 7.264; 120 h post-treatment, 2.643 ± 1.000; t = 3.401, p = 0.042) in partial bone metastatic foci (*n* = 7; 56 h pretreatment, 12.103 ± 7.792; 54 h post-treatment, 4.393 ± 1.786; t = 3.216, p = 0.049; 120 h pretreatment, 21.314 ± 11.169; 120 h post-treatment, 6.245 ± 2.239; t = 3.453, p = 0.014), and in multiple LN metastatic foci (*n* = 7; 56 h pretreatment, 9.854 ± 2.358; 54 h post-treatment, 3.346 ± 0.808; t = 6.536, p = 0.001; 120 h pretreatment, 13.125 ± 2.700; 120 h post-treatment, 3.861 ± 1.735; t = 9.143, p < 0.001) significantly reduced.

**Figure 5:**
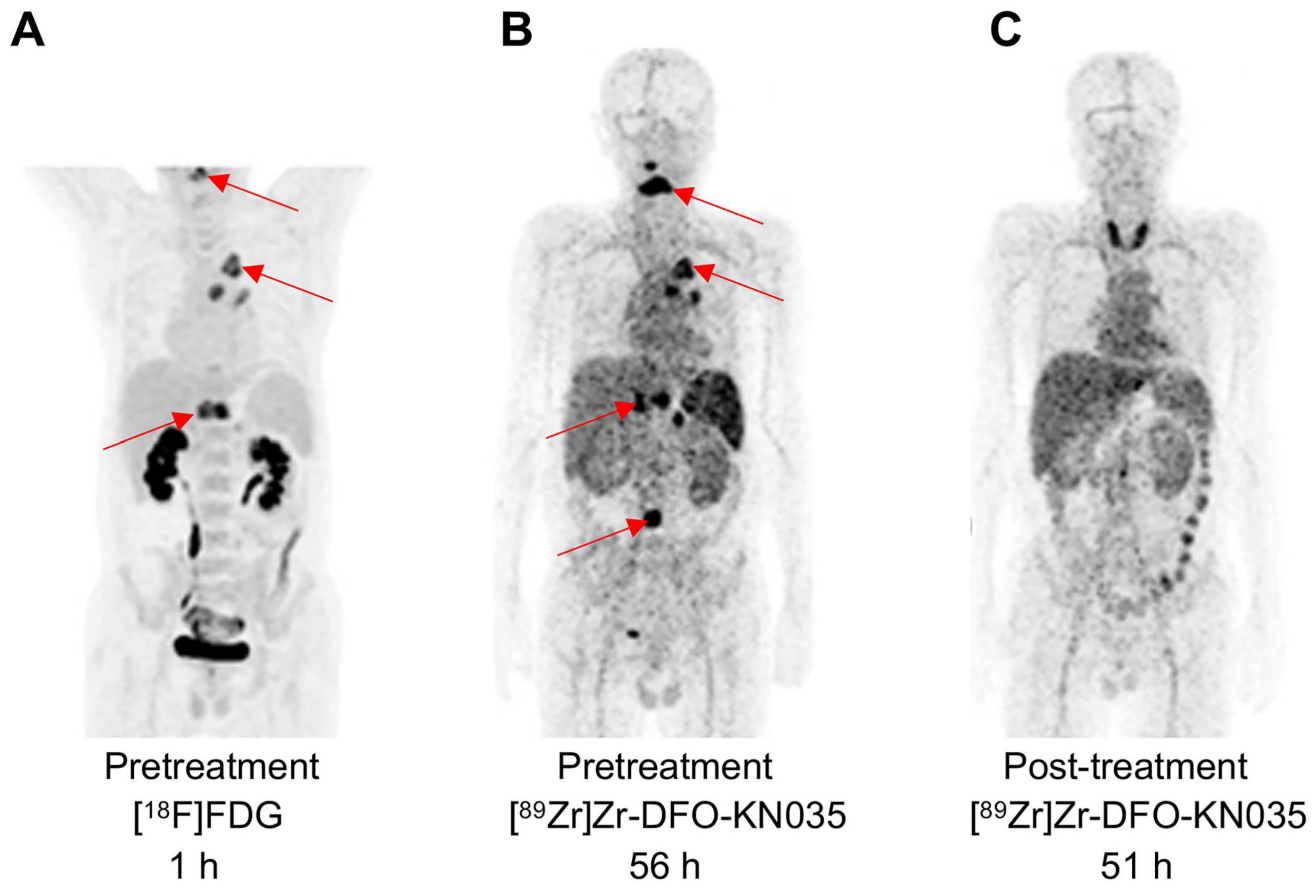
** PET imaging using [^89^Zr]Zr-DFO-KN035 before and after anti-PD-1 therapy of a patient (Case No.3) with PD-L1-positive lung cancer. (A)** MIP image of the patient acquired with the [^18^F]FDG tracer at 1 h before combined therapy including the anti-PD-1 drug sintilimab. **(B)** MIP image of the same patient obtained by using the molecular-imaging tracer [^89^Zr]Zr-DFO-KN035 at 56 h before anti-PD-1 treatment. **(C)** MIP image of the same patient obtained by using the molecular-imaging tracer [^89^Zr]Zr-DFO-KN035 at 51 h after anti-PD-1 treatment. The images show the reduction in radioactive uptake in and shrinkage of the cancer focus. Two cycles of treatment, 2 months.

As illustrated in **Figure 5A and Figure S7A**, pathological examination and [^18^F]FDG PET imaging identified a PD-L1 positive case (TPS = 60%) with primary lung cancer and multiple bone metastases. [^89^Zr]Zr-DFO-KN035 immunoPET imaging showed relatively high uptakes in the lung primary tumor and multiple bone metastases before treatment, and the radioactive uptakes decreased after 2 months of treatment (two cycles of combined sintilimab for anti-PD-1 therapy with a maximum dose of 200 mg) (**Figure 5B-C and Figure S7B**). This finding further suggested that [^89^Zr]Zr-DFO-KN035 PET has good imaging performance in patients with PD-L1-positive tumors and can enable the observational prediction of prognosis and monitoring of therapeutic efficacy. After anti-PD-1 treatment, high radioactive uptake occurred in the thyroid region. Serological testing after imaging revealed that this patient had low FT_3_, FT_4_, TT_3_ and TT_4_ but high TSH, 468.8 IU/ml TPOAb, and TgAb > 5000 IU/ml. This result suggested that [^89^Zr]Zr-DFO-KN035 immunoPET may have the potential to detect and predict important adverse effects such as immunologic thyroiditis in anti-PD-1 therapy.

**Figure 6:**
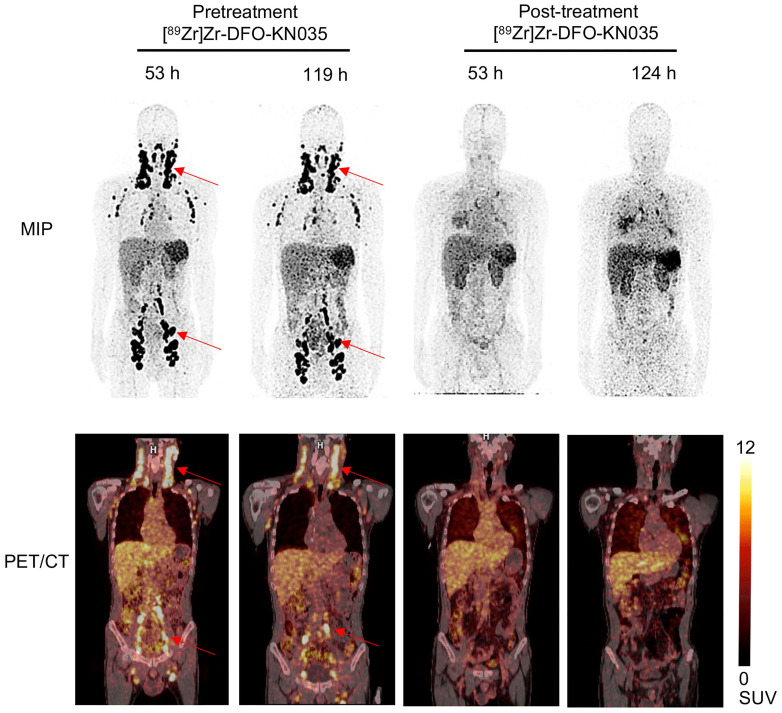
** Comparison of [^89^Zr]Zr-DFO-KN035 immunoPET imaging before and after treatment in patients with PD-L1-positive vascular lymphoma (Case No. 4).** MIP and coronal section of the PET/CT images of a PD-L1 positive (TPS = 30%) patient (malignant vascular lymphoma) acquired through [^89^Zr]Zr-DFO-KN035 immunoPET before (53 and 119 h) and after (53 and 124 h) combined anti-PD-1 therapy. The images show reductions in radioactive uptake at lymphoma focus sites at both time points. Red arrows show the locations of primary tumor and metastatic focus sites.

As indicated in **Figure 6**, in a PD-L1-positive (TPS = 30%) patient with refractory vascular lymphoma, [^89^Zr]Zr-DFO-KN035 immunoPET (scanning time-points of 53 and 119 h pretreatment) exhibited good imaging performance in detecting multiple LN tumor foci and high splenic uptake. Through the combination of chemotherapy and [^89^Zr]Zr-DFO-KN035 immunoPET scanning at two time points, the primary tumor lesions were found to have high radioactive uptakes before treatment. After 2 months of treatment, the radioactive uptakes in tumor foci dramatically reduced. Taken together, these results indicated that [^89^Zr]Zr-DFO-KN035 immunoPET had good efficiency for the imaging diagnosis of PD-L1-positive tumors, prediction of the prognosis of patients, and monitoring of the curative effect of antitumor treatment.

## Discussion

By screening clinical cases and conducting a comparative study with PET/CT imaging, we found that [^89^Zr]Zr-DFO-KN035 had specific targeting and high uptake in primary and metastatic tumor lesions in PD-L1-positive patients. However, these uptake values had no positive correlation with the expression level of PD-L1 in tumor samples. This lack of correlation may be attributed to several possible reasons. First, small sample sizes and different tumor types may lead to statistical bias. Second, a single tissue biopsy cannot correctly reflect the expression level of PD-L1 in different lesions. In addition, a time point difference exists between the biopsy detection and PET imaging. These factors may hinder obtaining a positive linear correlation between the uptake in PET imaging and positive rate of PD-L1 detected by IHC. In our study, [^89^Zr]Zr-DFO-KN035 immunoPET molecular imaging was sensitive in tumor-targeting imaging and could detect multiple metastatic lesions, such as multiple bone and LN metastases. In terms of nonradioactive toxicity, the antibody drug KN035 may have lower potential adverse reactions due to its low dosage. Moreover, [^89^Zr]Zr-DFO-KN035 immunoPET could monitor and predict the sites of side effects that may result from immunotherapy. [^89^Zr]Zr-DFO-KN035 immunoPET could provide imaging results reflecting therapeutic responses after therapies, including anti-PD-1 treatment. Such results can help in efficacy evaluation and patient-prognosis prediction.

PD-1 inhibits T cell activation and cytokine production by binding with its ligand PD-L1, thus playing a crucial role in maintaining peripheral tolerance 28-31. After activation, T cells express PD-1 receptor molecules on the membrane surface and produce interferons, which can also induce the expression of PD-L1 in tissues (including tumors) 32-35. Tumor cells and microenvironment limit immune response and cause immune escape by upregulating the expression and binding of PD-L1 to PD-1 on tumor-specific CD8+ T cells. By blocking the PD-1/PD-L1 signaling pathway, T cells can be intensively activated to recognize and kill tumor cells, resulting in the effective inhibition of tumor growth, causing PD-L1 to become a promising target of tumor immunotherapy. Therefore, the inhibition of PD-1/PD-L1 signaling and blocking of tumor immune escape have become a hotspot in antitumor treatment. Although blocking PD-1/PD-L1 signaling can improve antitumor immunity and achieve good efficacy in the treatment of various tumors 36, in the absence of early screening, the response rate to treatment is generally only 15%-20% 37-39. However, in PD-L1-positive patients, the response rate of anti-PD-L1 treatment rises to approximately 50% 34. PD-L1 detection by IHC is the gold standard for the screening of patients who could benefit from anti-PD-L1 therapy. However, this method is limited by its inabilities to provide non-invasive, real-time, and comprehensive detection results and inability to give help in monitoring the occurrence and real-time state of adverse reactions. The [^89^Zr]Zr-DFO-KN035 immunoPET imaging technique used in this study can provide noninvasive and comprehensive monitoring results and predict the likely sites of adverse responses (such as thyroiditis) on the basis of radioactivity distribution in imaging.

PD-L1 is highly expressed in numerous solid malignancies. It also promotes the growth of tumor cells and induces the apoptosis of lymphocytes 40, which is related to the poor prognosis of tumors. Inhibiting PD-1/PD-L1 signaling by using anti-PD-L1 antibody drugs restores immune function and exerts excellent antitumor effects on various lymphomas and solid tumors. In A549 cells, PD-L1 downregulation inhibits proliferation and induces apoptosis, whereas in non-small-cell lung carcinoma, PD-L1 overexpression indicates high invasiveness and poor prognosis 41, 42. The prognosis of 34 cases of patients with renal clear cell carcinoma and high PD-L1 expression is poor, and PD-L1 is also expressed in primary foci and tumor-infiltrating lymphocytes 43. High PD-L1 expression in tumor cells or tumor-infiltrating lymphocytes is positively correlated with tumor late staging, low survival rate, and rapid metastasis. Given the poor correlation between PD-L1 expression levels in primary and metastatic renal cell carcinoma, PD-L1 expression cannot be used as a theoretical basis for selecting PD-L1 inhibitors for patients with metastatic renal cell carcinoma. PD-L1 expression in tumors cannot be determined through single-site biopsy because of the heterogeneity of tumor tissues. Despite conflicting results, PD-1/PD-L1 signaling inhibitors have been proven to be effective as a novel immunotherapy for solid tumors such as lung cancer 44. PD-L1 expression may be associated with the effectiveness of anticancer immunotherapy 45. Therefore, the accurate and comprehensive molecular detection of PD-L1 is highly important for antitumor-immunotherapy selection and cancer prognosis prediction.

PET molecular imaging using targeting molecules, such as radionuclide-labeled antibodies, have many diagnostic advantages over conventional imaging methods and pathological biopsies. Molecular radionuclide imaging is directly related to disease and can also provide the *in vivo*, continuous, multidimensional, structural, and functional evaluation of drug therapy. With molecular imaging techniques, the therapeutic effect of drugs can be directly observed in humans or animals and can be applied to screen patients who may benefit from antitumor immunotherapy. The radionuclide zirconium-89 is an ideal choice for high-resolution PET imaging and is extensively used in antibody drug radiolabeling and PET imaging 21, 46. Pembrolizumab (a commercial anti-PD-1 antibody, Keytruda Merck) has been used in clinical trials to treat non-small-cell lung carcinoma 47. ^89^Zr-labeled anti-PD-L1 antibodies including atezolizumab (anti-PD-L1 antibody, Tecentriq Roche), have also been used in clinical trials on advanced solid tumors and diffuse large B-cell lymphoma. Consistent with our study, previous works found that these PD-L1-targeting tracers showed good efficiency in visualizing tumor lesions and had no tracer-related adverse effects 48-50.

KN035, an anti-PD-L1 antibody, is the first monodomain antibody for tumor immunotherapy in clinical research and the first antitumor immunotherapy antibody applied through subcutaneous injection 20. KN035 has been reported to the FDA, NMPA, and PMDA for multicenter clinical trials and is used for the therapy of solid tumors including hepatocellular carcinoma 25, 51-52. Two phase I clinical trials (NMPA clinical registration number: CTR20170036 and CTR20191855; used for sepsis and solid tumors, at a dosage of 0.1-10 mg/kg), two phase II clinical trials (CTR20181127 and CTR20181124; for colorectal cancer and stomach cancer at 150 mg once a week or 5 mg/kg twice a week), and one phase III clinical trial (CTR20180332; for biliary tract carcinoma, at 2.5 mg/kg once a week) on KN035 have been initiated in China. KN035 has achieved important breakthroughs in expected efficacy, safety, preparation and dosage form development, and precision medicine. The results of clinical trials have shown that KN035 has no serious adverse events or drug-induced allergic reactions. Preliminary research has demonstrated that in animal model studies, the diagnostic antibody drug [^89^Zr]Zr-DFO-KN035 has high and long-lasting uptake in tumors 21. For clinical trial application, micro-PET/CT scanning was also performed on tumor-bearing mice. Radioactive substance uptake in tumors also showed a continuous upward trend after [^89^Zr]Zr-DFO-KN035 injection. Bone uptake increased slowly at 12 h and peaked at 97 h; kidney uptake peaked at 2.5 h and then decreased; brain tissue uptake decreased slowly; and liver uptake decreased during 1-24 h and increased slightly at 52 and 97 h. A study on [^89^Zr]Zr-DFO-KN035 biodistribution demonstrated that in cynomolgus monkeys, the heart, liver, and kidney showed obvious radioactivity after injection 21. Lung radioactivity was observed only at the initial hours after injection and decreased to background levels 1 day later. In the heart, the radioactive tracer concentration was highest at 2 h after injection and uptake decreased at 72-169 h. The uptake of radioactive substances in the liver did not change considerably within 2-49 h after injection, and uptake in the kidneys peaked at 72 h after injection and decreased gradually at the subsequent time points. We performed experiments on mice and guinea pigs to study abnormal toxicity. No death occurred during the experiments, and body weight increased indiscriminately compared with that in the control group, thereby meeting test requirement. On the basis of previous data, we believe that the clinical use of [^89^Zr]Zr-DFO-KN035 has low expected risks and that possible adverse responses can be controlled.

The common adverse reactions of immunotherapy are skin reaction, gastrointestinal reaction, liver injury, immune-related pneumonia, thyroid dysfunction and thyroiditis, hypophysitis, and adrenal hypofunction. In this study, [^89^Zr]Zr-DFO-KN035 immunoPET showed high uptake in the thyroid, suggesting the greate accumulation of PD-L1 antibodies in this organ. The accumulation in the thyroid may be related to adverse reactions, such as thyroiditis in tumor immunotherapy with anti-PD-L1 drugs. Therefore, [^89^Zr]Zr-DFO-KN035 immunoPET may have a potential effect on predicting adverse events in immunotherapy.

In the present work, we used the results of PD-L1 pathological examination for patient screening in accordance with criteria (TPS>1%, PD-L1-positive cases; TPS>50%, PD-L1 high-expression cases) 26, 27 to analyze the efficiency of [^89^Zr]Zr-DFO-KN035 immunoPET in noninvasive, *in vivo*, and tumor diagnostic imaging and conducted a comparative radiographic analysis with [^89^Zr]Zr-DFO-KN035 in patients with tumors. Before antitumor treatment, we compared and analyzed the differences and advantages of the [^18^F]FDG PET and [^89^Zr]Zr-DFO-KN035 immunoPET diagnostic techniques. [^89^Zr]Zr-DFO-KN035 immunoPET showed no imaging features in the PD-L1-negative case and high uptakes at tumor sites in positive cases. These results demonstrated the specificity and sensitivity of the molecular-imaging tracer [^89^Zr]Zr-DFO-KN035 to PD-L1 *in vivo*. Compared with [^18^F]FDG PET, [^89^Zr]Zr-DFO-KN035 immunoPET had a slightly higher sensitivity and could detect numerous metastatic lesions, and the tumor-specific uptake of [^89^Zr]Zr-DFO-KN035 was similar to that of [^18^F]FDG. [^89^Zr]Zr-DFO-KN035 demonstrated good imaging efficiency for various tumor types with or without the comparison of [^18^F]FDG. Comparison with PET/CT imaging before and after antitumor treatment revealed that [^89^Zr]Zr-DFO-KN035 immunoPET could also intuitively and clearly show imaging differences and therapeutic efficacy. However, [^89^Zr]Zr-DFO-KN035 showed some deficiencies as a diagnostic radiographic tracer. For example, PD-L1 expressed in normal tissues also led to high radioactive uptakes. Therefore, although [^89^Zr]Zr-DFO-KN035 immunoPET can be used in the noninvasive and comprehensive differentiation of patients with PD-L1-positive or negative tumors to guide precise antitumor therapy, it cannot be used in the diagnosis and efficacy monitoring of PD-L1-negative patients.

## Conclusions

We demonstrate that the anti-PD-L1 monodomain antibody KN035 has the potential for the diagnosis and therapeutic evaluation of various tumors, including solid tumors and hematological system tumors. It has high value in clinical development for the diagnosis and treatment of major malignancies, such as lung and liver cancers. The results of previous animal experiments and clinical studies revealed that KN035 has good safety and controllable low risk. In this study, we demonstrated the targeting of PD-L1-positive human tumors by [^89^Zr]Zr-DFO-KN035 through *in vivo* PET imaging and analyzed and verified the imaging efficiency and advantages of the molecular imaging tracer. Furthermore, the results of our study may be of great guiding value for the follow-up clinical research on KN035 as a diagnostic and therapeutic drug carrier for malignant tumors.

## Supplementary Material

Supplementary figures.

## Figures and Tables

**Table 1:** Characteristics and treatments of the included clinical cases.

Patient	M/F	Age (y)	Tumor type	PD-L1 expression TPS (%)	Treatment	Radio-dose (MBq) pre-/post-treatment	[^89^Zr]Zr-DFO-KN035 immunoPET/CT (h) pre-/post-treatment
1	M	59	Lung cancer with lymph node, bone and brain metastasis	40	Pemetrexed, Carboplatin, Sintilimab	pre-, 59.20; post-, 73.26	pre-, 20, 56, 115; post-, 55, 120
2	M	70	Lung cancer with multiple bone metastasis	40	Paclitaxel, Carboplatin	pre-, 75.85	pre-, 54, 100
3	M	51	Lung cancer with bone metastasis	60	Excision, Paclitaxel, Carboplatin, Sintilimab	pre-, 86.21; post-, 86.58	pre-, 54, 120; post-, 51, 118
4	M	58	Malignant lymphoma	30	Cyclophosphamide, Doxorubicin, Vincristine, Etoposide	pre-, 84.36; post-, 87.69	pre-, 53, 119; post-, 53, 124
5	M	54	Lung cancer with metastasis	20	Pemetrexed, Carboplatin	pre-, 82.51	pre-, 51, 123
6	M	77	Lung cancer with lymph node metastasis	70	Pemetrexed, Sintilimab	pre-, 75.85	pre-, 56, 124
7	F	37	Nasopharyngeal carcinoma	40	Docetaxel, Nedaplatin	pre-, 88.43	pre-, 53, 124
8	F	53	Colorectal cancer with lymph node metastasis	15	Anlotinib, Penpulimab	pre-, 83.99	pre-, 52, 124
9	M	58	Nasopharyngeal carcinoma with lymph node metastasis	60	Docetaxel, Nedaplatin, Nimotuzumab	pre-, 81.03	pre-, 56, 120
10	F	63	Malignant melanoma of right lung, Secondary malignant tumor of cervical lymph node	70	Toripalimab	pre-, 77.33	pre-, 56, 123
11	F	56	Colorectal cancer with liver metastasis	<1	Anlotinib, Sintilimab	pre-, 86.95	pre-, 54, 120

**Table 2:** Number of lesions found through PET/CT with the two tracers.

PET/CT	Primary lesions	LN metastases	Bone metastases	Other lesions
[^18^F]FDG	1.0 ± 0.0	5.8 ± 4.7	1.8 ± 1.6	2.2 ± 3.9
[^89^Zr]Zr-DFO-KN035	1.0 ± 0.0	4.6 ± 3.7	1.9 ± 2.5	0.7 ± 1.2

**Table 3:** Tumor PD-L1 expression (IHC) and tracer uptake in [^89^Zr]Zr-DFO-KN035 immunoPET.

PD-L1 expression TPS (%)	Primary focus uptake at 54 h		Visualized number of metastases
	target-to-muscle ratio of SUVmax	Target-to-muscle ratio of SUVmean	
40	2.10	1.49	9
40	4.49	2.58	6
60	8.03	4.87	12
30	20.50	10.42	15
20	4.74	2.97	9
70	8.02	5.24	7
40	4.95	2.81	4
15	14.09	10.08	9
60	5.57	3.49	7
70	6.91	4.25	4

## Abbreviations

CT: computed tomography
IHC: immunohistochemistry
immunoPET: immuno-positron emission tomography
KN035: a recombinant humanized PD-L1 monodomain antibody Fc fusion protein
LN: lymph node
mAbs: monoclonal antibodies
PD-1: programmed cell death receptor-1
PD-L1: programmed cell death receptor ligand-1
PET: positron emission tomography
SUV: standard uptake value
TBR: target-to-background ratio
TPS: tumor cell proportion score
