# Supplementary material for: Imaging diagnosis and efficacy monitoring by [89Zr]Zr-DFO-KN035 immunoPET in patients with PD-L1-positive solid malignancies
Source: Theranostics. 2024 Jan 1;14(1):392–405. doi: 10.7150/thno.87243 (PMC10750192; doi:10.7150/thno.87243)
Supplement: Supplementary file 1 — Supplementary figures. [file thnov14p0392s1.pdf]

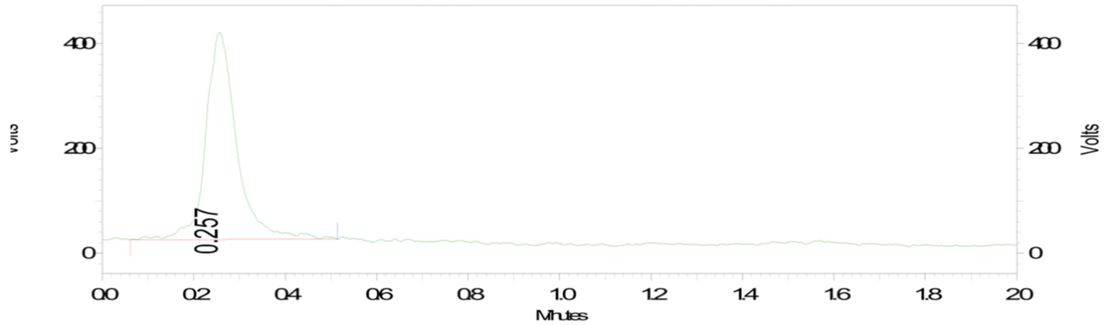


**Figure S1. iTLC of [$^{89}$Zr]Zr-DFO-KN035 radiolabeling.**

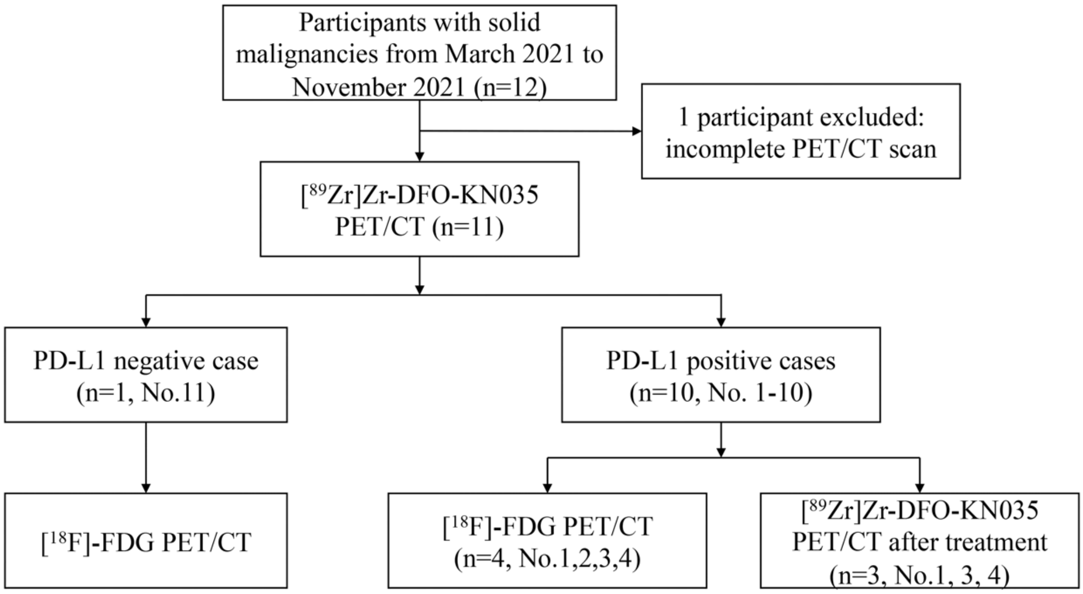


**Figure S2. Study Graphset tables.**

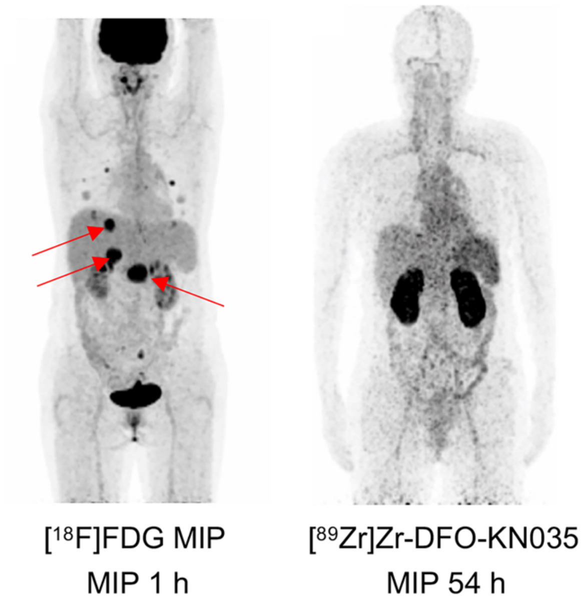


**Figure S3. Comparison of the [$^{18}$F]FDG and [$^{89}$Zr]Zr-DFO-KN035 PET imaging.** MIP images of a PD-L1-negative patient (colorectal cancer and liver metastasis) obtained with the PET tracers [$^{18}$F]FDG (scanned at 1 h after injection) and [$^{89}$Zr]Zr-DFO-KN035 (54 h).

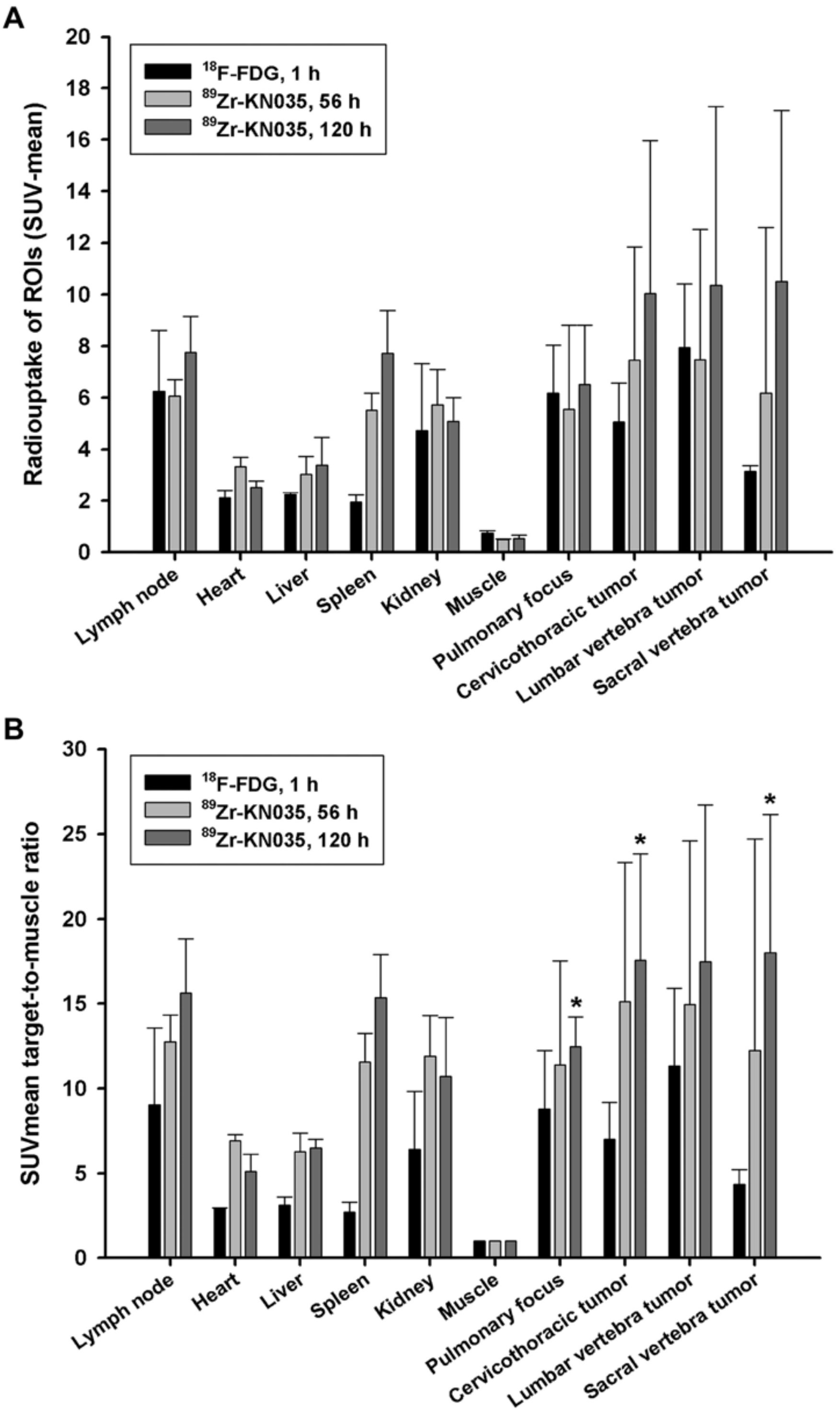


**Figure S4. Comparative analysis of two PET/CT methods for the imaging diagnosis of primary lung cancer and multiple metastases in PD-L1-positive patients. (A)** Statistical chart of radioactive uptake values (SUVmean) at different regions of interest (ROIs) in the [$^{18}$F]FDG (1 h) and [$^{89}$Zr]Zr-DFO-KN035 (56 and 120 h) PET imaging of three PD-L1 positive patients with

lung primary cancer and multiple metastases. Data are expressed as the mean ± SD, and $n = 3$ for each site. **(B)** Statistical chart of the target-to-background ratios of SUVmean ([$^{18}$F]FDG 1 h and [$^{89}$Zr]Zr-DFO-KN035 56 and 120 h) of different ROIs of tissues (background tissue and muscle). Data are expressed as the mean ± SD, and $n = 3$ for each site. One-way ANOVA was used and, $^{*}p < 0.05$ vs. [$^{18}$F]FDG (1 h) PET group.

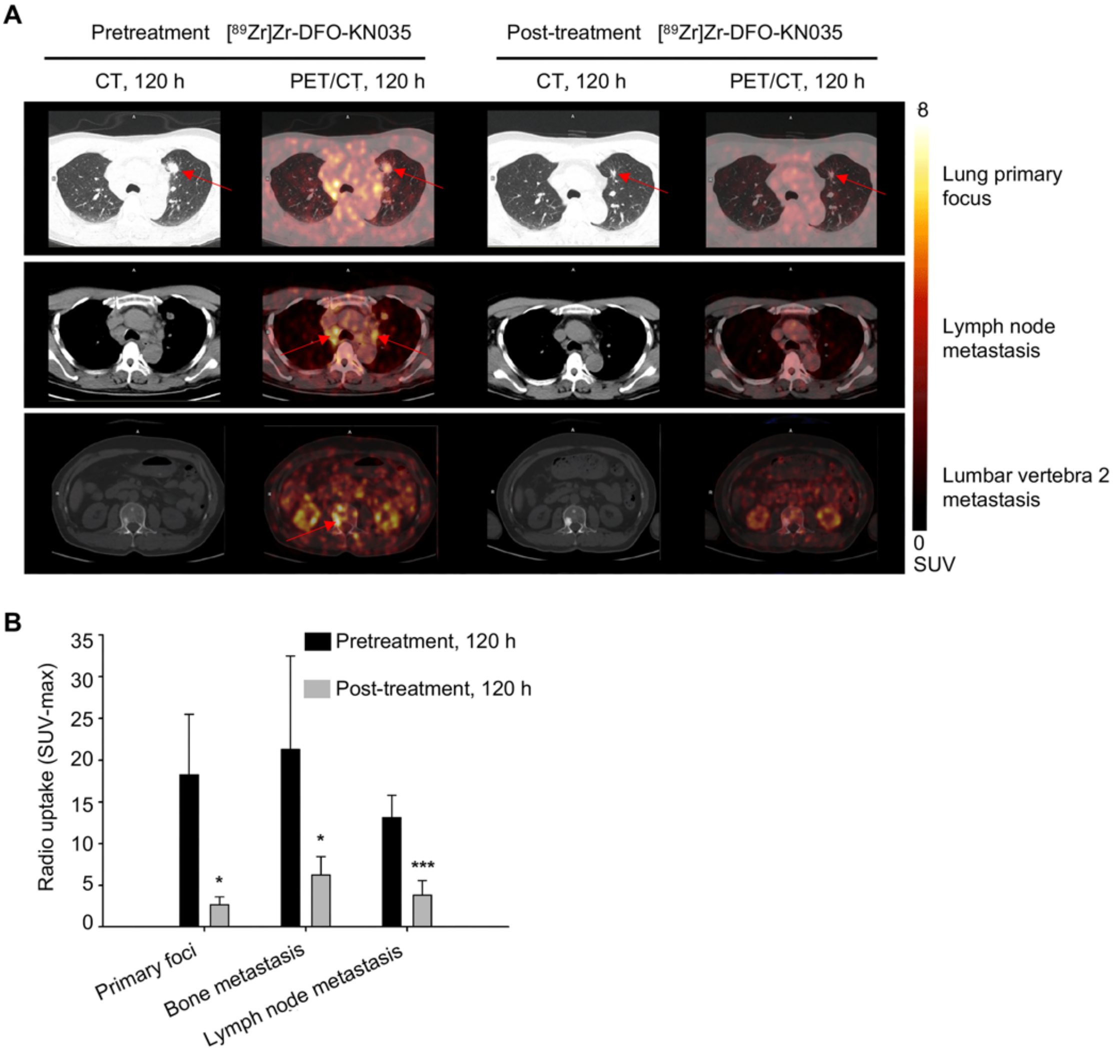


**Figure S5. Comparison of primary tumors and metastases in PD-L1-positive patients using [$^{89}$Zr]Zr-DFO-KN035 immunoPET before and after treatment. (A)** Representative **(Case No.1)** CT and PET/CT images of the layers of primary focus (first row), lymph node metastasis (second row), and bone metastasis (third row, lumbar vertebra 2) sites in the same patient with PD-L1-positive cancer (lung primary cancer with bone, lymph node, and brain metastasis) obtained by using [$^{89}$Zr]Zr-DFO-KN035 before (120 h) and after (120 h) treatment. Red arrows show the locations of primary tumor and metastasis focus sites. **(B)** Statistical chart and comparison of the radioactive uptakes (SUVmax) of the primary and metastatic tumors in [$^{89}$Zr]Zr-DFO-KN035 immunoPET imaging before (120 h) and after (120 h) treatment. Data are expressed as the mean ±

SD, and $n = 3$–7 for each site. Paired samples t-test was conducted. *$p < 0.05$, **$p < 0.01$, and ***$p < 0.001$ vs. pretreatment group.

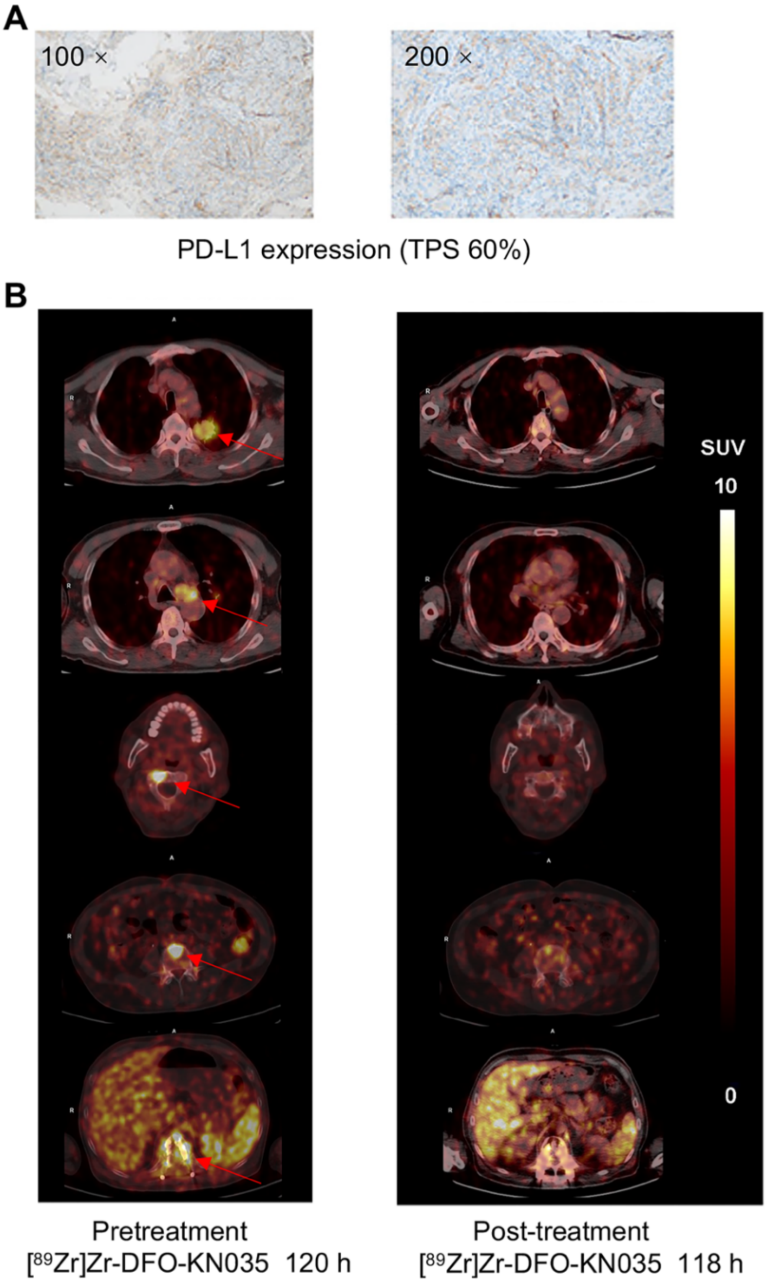


**Figure S6. PET imaging using [$^{89}$Zr]Zr-DFO-KN035 before and after anti-PD-1 therapy of a patient (Case No.3) with PD-L1-positive lung cancer. (A)** Immunohistochemical result of a PD-L1 positive patient (TPS = 60%) with lung cancer and multiple bone metastases. **(B)** Representative PET/CT images of the layers of lung primary cancer (first lane) and some bone metastatic sites (lower lanes) in the same patient taken by using [$^{89}$Zr]Zr-DFO-KN035 at pre- (120 h) and post-treatment (118 h).

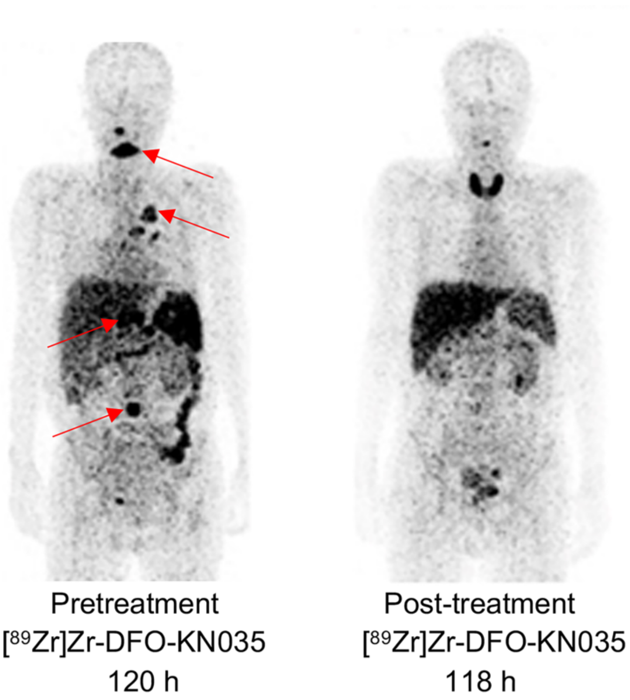


**Figure S7. PET imaging using [$^{89}$Zr]Zr-DFO-KN035 before and after anti-PD-1 therapy of a patient (Case No.3) with PD-L1-positive lung cancer. (A)** MIP image of a PD-L1 positive patient (TPS = 60%) with lung cancer and multiple bone metastases. **(B)** MIP images of the patient obtained by using the molecular-imaging tracer [$^{89}$Zr]Zr-DFO-KN035 before (120 h post-injection) and after (118 h post-injection) anti-PD-1 treatment.
